# A novel unbiased counting method for the quantification of synapses in the mouse brain

**DOI:** 10.1016/j.jneumeth.2014.10.020

**Published:** 2015-01-30

**Authors:** Florian Reichmann, Evelin Painsipp, Peter Holzer, Daniel Kummer, Elisabeth Bock, Gerd Leitinger

**Affiliations:** a Research Unit of Translational Neurogastroenterology, Institute of Experimental and Clinical Pharmacology, Medical University of Graz, Universitätsplatz 4, 8010 Graz, Austria; b Research Unit Electron Microscopic Techniques, Institute of Cell Biology, Histology and Embryology, Medical University of Graz, Harrachgasse 21, 8010 Graz, Austria

**Keywords:** DCV, dense-core vesicles, DGgl, dentate gyrus, granular cell layer, DGpl, dentate gyrus, polymorph cell layer, EE, environmental enrichment, MWM, Morris water maze, NPY, neuropeptide Y, Disector, Mouse, Environmental enrichment, Electron microscopy, Dentate gyrus, Dense core vesicle

## Abstract

**Background:**

The numerical density of synapses and their ultrastructural features are best assessed with electron microscopy. Counting is done within counting frames placed on a pair of sections (disector technique). But this requires that the thin sections are taken from comparable brain regions and the disectors are placed in a uniform random fashion. Small brain areas like the polymorph layer of the mouse dentate gyrus are difficult to encounter, and manually moving the microscope stage for placing the micrographs seems arbitrary.

**New method:**

Here the polymorph layer was approximated with 20 μm thin, Nissl-stained vibratome sections. The subsequent vibratome section was processed for electron microscopy and serially thin sectioned. The microscope stage was moved using a random number generator, placing at least 20 disectors onto a pair of sections. The numerical synapse density, the numerical density of dense-core vesicles, and other ultrastructural features were compared between mice that had been kept in an enriched environment and mice kept under standard housing conditions.

**Results:**

Environmental enrichment significantly decreased the numerical density of dense-core vesicles and synaptic cleft widths within the polymorph layer, associated with behavioral improvement in the Morris water maze, a hippocampus-dependent task of spatial learning and memory.

**Comparison with existing methods:**

This procedure was easy to handle and enabled us to produce thin sections in small, defined brain areas. Furthermore, placing the disectors with random numbers excluded observer bias.

**Conclusions:**

Our procedure provides an uncomplicated way of assessing numerical densities in small brain areas in an unbiased manner.

## Highlights

• We describe a novel method for synapse quantification using electron microscopy.
• Nissl-stained vibratome sections allowed accurate brain region identification.
• Automatic microscope stage shifts using custom-made software excluded observer bias.
• The method showed altered synaptic features after environmental enrichment.

## Graphical abstract

**Figure:**
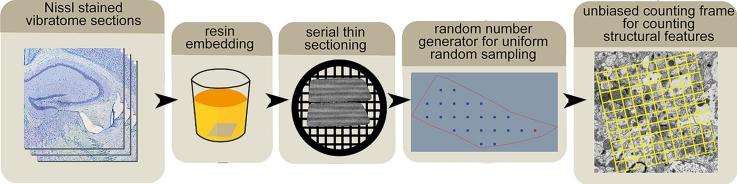

## 1 Introduction

Synapses are key mediators of communication between neurons, and several of their ultrastructural features can be determined electron microscopically, which allows deductions as to neuronal activity. For example the numerical density of synapses, sizes of the active zones and of the postsynaptic densities, the widths of the synaptic clefts, the numbers of small clear vesicles that are docked at the active zones and the numbers of those small, clear vesicles which are undocked, but associated with an active zone give useful hints as to synaptic activity (Nakamura et al., 1999). However, small clear vesicle release at synapses is not the only way in which neurons communicate with each other. Neuropeptides are major regulators of brain function (Nurrish, 2014) and they can have an influence on mood, anxiety, or social interactions (Garrison et al., 2012; Kormos and Gaszner, 2013; Walker and McGlone, 2013). These peptides are generally stored in, and released from, large dense-core vesicles (DCV) in neurons, where they co-exist with classical neurotransmitters (Merighi et al., 2011). Determining the numerical density of DCV could thus allow deductions as to how much neuropeptide is stored in a particular brain area.

Using microscopy, the numerical densities of objects are most efficiently assessed with a disector (Sterio, 1984; Gundersen et al., 1988). A disector consists of a pair of serial sections taken at a known interval. If a particles’ transect is seen on one section (the reference section), but not on the other (the lookup section), it is counted. An unbiased counting frame denotes boundaries of the area in which the particles are counted within the plane of the reference and lookup sections, and objects that extend across two forbidden lines on the counting frame are omitted from the count (de Groot and Bierman, 1986). For electron microscopy (EM), a disector can consist of two thin sections in a series.

In order to obtain an unbiased estimate of numerical densities, it is necessary to keep to a systematic random sampling protocol (Howard and Reed, 2004), so that every part of the structure under investigation has the same chance of being included in the disectors. If a particular brain region is to be investigated, this region needs to be identified by an expert neuroanatomist, dissected, and processed for EM. High accuracy in identifying the particular brain region is very important so that comparable regions are investigated in every sample. Furthermore, when the reference and the lookup section are being investigated under the electron microscope, the counting frames need to be placed within the area of interest in a uniform random way. Systematic random sampling for EM studies of the brain therefore requires both accurately finding a particular brain region and then placing disectors into this region onto randomly determined positions.

The aim of this study was to test a novel unbiased approach to assess ultrastructural synaptic parameters in the mouse brain and to evaluate its potential to reveal synaptic alterations in brains of mice kept under environmental enrichment (EE). Enriched housing is a way to enhance the welfare of laboratory animals by keeping them in a species-specific environment. Compared with housing under standard laboratory conditions, EE improves cognition, emotional-affective behavior, learning and memory (Simpson and Kelly, 2011). Several mechanisms have been associated with these behavioral changes including neurochemical as well as structural brain alterations. In experimental animals maintained under EE, EM has been shown to be a valuable tool to assess structural changes at the synaptic level. Various studies reported EE-induced changes of the synaptic ultrastructure dependent on the brain region examined and the enrichment paradigm used (Nakamura et al., 1999; Rampon et al., 2000; Xu et al., 2009; Landers et al., 2011).

Here, we combined behavioral experiments with an ultrastructural study to relate the effect of EE on cognitive performance with that on synaptic ultrastructure in mice. A novel unbiased approach was used to assess ultrastructural synaptic parameters in the polymorph cell layer, a sublayer of the dentate gyrus (DGpl) (Amaral et al., 2007). The dentate gyrus was selected as previous studies reported strong effects of EE on adult neurogenesis in this brain area (Kempermann et al., 1997; Schloesser et al., 2010; Tanti et al., 2012). The main reason to investigate the DGpl within the dentate gyrus was that preliminary results demonstrated increased neuropeptide Y (NPY) expression in this cell layer after EE (Reichmann et al., 2012). A further reason was that EE also increases the activity of dentate granule cells, as measured by the expression of the immediate early gene c-Fos (Reichmann et al., 2013), which may result in EE-evoked enhanced signaling because some granule cells terminate in the DGpl (Acsady et al., 1998).

For this new method, we used Nissl-stained vibratome sections of only 20 μm thickness through the mouse brain to identify the dentate gyrus with high accuracy. Furthermore, uniform random sampling was performed by automatically controlling the microscope stage to move to stage shifts that had previously been determined with a random number generator. We determined several ultrastructural parameters within the DGpl, and we demonstrate that our approach is useful to show that the width of the synaptic clefts and the numerical density of DCV significantly changed in the polymorph cell layer during housing with EE. On the following pages we provide a detailed protocol how to investigate this structure by electron microscopy in a systematic and reliable way.

## 2 Materials and methods

### 2.1 Experimental animals

Twenty female C57BL/6J mice, aged 16 weeks, obtained from the Division of Laboratory Animal Science and Genetics of the Department of Biomedical Research of the Medical University of Vienna (Himberg, Austria) were used for the Morris water maze (MWM) experiment. For the EM experiment ten female C57BL/6N mice of the same age obtained from Charles River (Sulzfeld, Germany) were used as the previous source of animals was no longer available at the start of this experiment. Mice were kept in the in-house animal facility under controlled conditions of temperature (set point 21 °C) and air humidity (set point 50%) and under a 12 h light/dark cycle (lights on at 5:30 h, lights off at 17:30 h). All experiments were approved by an ethical committee at the Federal Ministry of Science and Research of the Republic of Austria (BMWF-80.104/2-BrGT/2007 and BMWF-66.010/0037-II/3b/2013) and conducted according to the Directive of the European Communities Council of 24 November 1986 (86/609/EEC) and the Directive of the European Parliament and of the Council of 22 September 2010 (2010/63/EU).

### 2.2 Environmental enrichment

Animals were housed under either standard or EE conditions for 5 weeks. This differential housing procedure was carried out as previously described (Reichmann et al., 2013). Briefly, standard-housed mice were kept in small polycarbonate cages (36.5 cm × 20.7 cm × 14.0 cm; length × width × height) in groups of 5, while EE-housed mice were kept in large polycarbonate cages (59.0 cm × 38.0 cm × 20.0 cm; length × width × height). Additionally, EE-housed mice were provided with a running wheel, a hay tunnel and nesting material for the duration of the experiment. Furthermore, additional hard paper or polycarbonate tunnels and mouse houses were provided and exchanged on a weekly basis. This procedure corresponds to the last 5 weeks of the enrichment paradigm used in Reichmann et al. (2013).

### 2.3 Morris water maze

The MWM test was used to evaluate spatial learning and memory. The test was performed as previously described (Reichmann et al., 2013). Briefly, the MWM consisted of a hidden escape platform in an open circular tank made of black plastic material (diameter: 120 cm, depth: 60 cm) which was filled approximately half with water of 25–26 °C. The water in the tank was made opaque by addition of white non-toxic tempera paint (Rhoximat SB112, Rhodia, Paris, France). The task of a mouse placed in the water tank was to find the escape platform (diameter: 10 cm) that was submerged 1 cm below the water surface. The time needed to reach the platform (latency) was recorded with video tracking software (VideoMot2; TSE Systems, Bad Homburg, Germany). The entire MWM test procedure took five consecutive days, each animal being subjected to 6 trials per day with a minimum inter-trial interval of 30 min. During the first 3 days of the MWM test the hidden platform was placed in the south-east quadrant of the tank. This initial acquisition task was followed by the reversal task on days 4 and 5, during which the platform was positioned in the north-west quadrant of the tank.

### 2.4 Electron microscopy

Mice used for the EM experiment were euthanized with an overdose of pentobarbital (150 mg/kg IP). To prepare tissues for EM, the brains of five EE-housed and five standard-housed animals were dissected, and cut in half at the midline; one half was immediately immersed in fixative solution. Brains were fixed for two days in 2% formaldehyde, 2.5% glutardialdehyde in 0.1 M cacodylate buffer pH 7.4 at 4 °C and rinsed for at least 24 h in the same buffer at 4 °C.

**Fig. 1:**
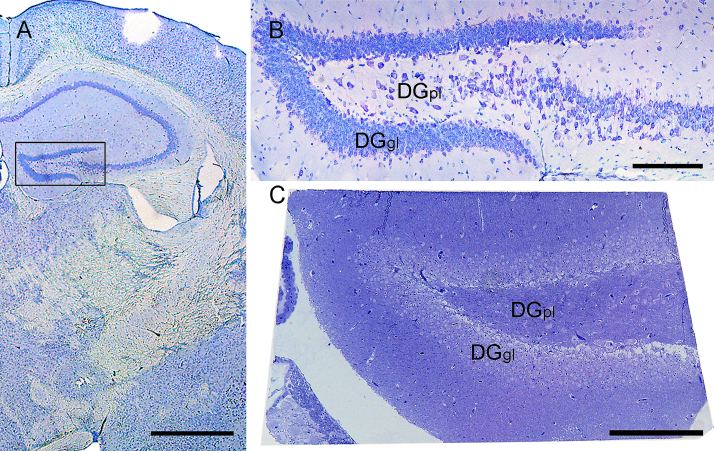
Stainings used to locate a specific level of the dentate gyrus. Panels A and B represent 20 μm-thick coronal vibratome sections of a mouse brain stained with thionine acetate close to Bregma −1.3. (A) Overview. The box shows the dentate gyrus. (B) Detailed view. (C) Semithin (500 nm thick) section of the dentate gyrus in the same orientation stained with toluidine blue. Scale bars 1 mm in A, 200 μm in B and C. DGgl, dentate gyrus, granular cell layer; DGpl, dentate gyrus, polymorph cell layer.

The DGpl was selected as the region of interest and identified by using coronal vibratome sections and the mouse brain atlas of Franklin and Paxinos (2008). To this end, the brain was trimmed using a vibratome (Leica VT 1000, Leica Microsystems, Vienna, Austria) close to Bregma −1.3. Coronal vibratome sections were sequentially taken at 20 μm thickness and Nissl-stained (Fig. 1A and B) using 0.05% thionine acetate (Ceristain, Merck, Darmstadt, Germany) in sodium acetate buffer, pH 4.2 for 1 min each. Each section was compared with the mouse atlas until the Bregma level −1.34 was reached. The use of these thin stained vibratome sections together with the mouse brain atlas permitted reliable and reproducible identification of the area of interest for every mouse brain used in the experiment. The next section was cut at 150 μm thickness and a small piece of the section containing the DGpl was microdissected with a razor blade. This part was post fixed for two hours in 2% osmium tetraoxide and embedded for EM in TAAB embedding resin (TAAB, Berkshire, UK) in a silicone flat embedding mold.

For the analysis of synapses and synaptic parameters, the block was trimmed and 55 nm thick serial thin sections were cut from the block surface with a Leica Ultracut UCT ultramicrotome (Leica Microsystems, Wetzlar, Germany), mounted on a slot grid covered with pioloform and contrasted with 2% uranyl acetate and lead citrate. Afterwards a 0.5 μm thick coronal semithin section was cut and stained with 0.5% toluidine blue solution for verifying that the thin sections contained the DGpl (Fig. 1C).

EM was performed with a Tecnai G2 20 electron microscope (FEI, Eindhoven, Netherlands) operated at 120 kV equipped with a Megaview wide angle camera (Olympus Soft Imaging Solutions, Münster, Germany) and a US 1000 digital camera (Gatan, Pleasanton, USA). For analysis, at least 20 image pairs were taken from two consecutive sections per animal.

Several software packages were used: TEM Imaging Analysis Software (TIA 4.7 SP1, FEI, Eindhoven, Netherlands) to obtain images, FEI Serial Section Software (FEI, Eindhoven Netherlands) to store the borders of consecutive sections and to move the stage from a position at one section to the corresponding position at the next section, FEI Montage software to obtain and merge adjacent camera images, and ImageJ (National Institutes of Health, USA) to analyze the images. In order to obtain image pairs that are distributed in a systematic random fashion, a custom-made software was written using Java script. FEI TEM Scripting was used as an interface to obtain the stage shift position and to control the stage shifts.

First, the borders of two consecutive sections were entered in FEI Serial Section software, following the instructions of the software, so that corresponding locations between the first and second section could be found.

**Fig. 2:**
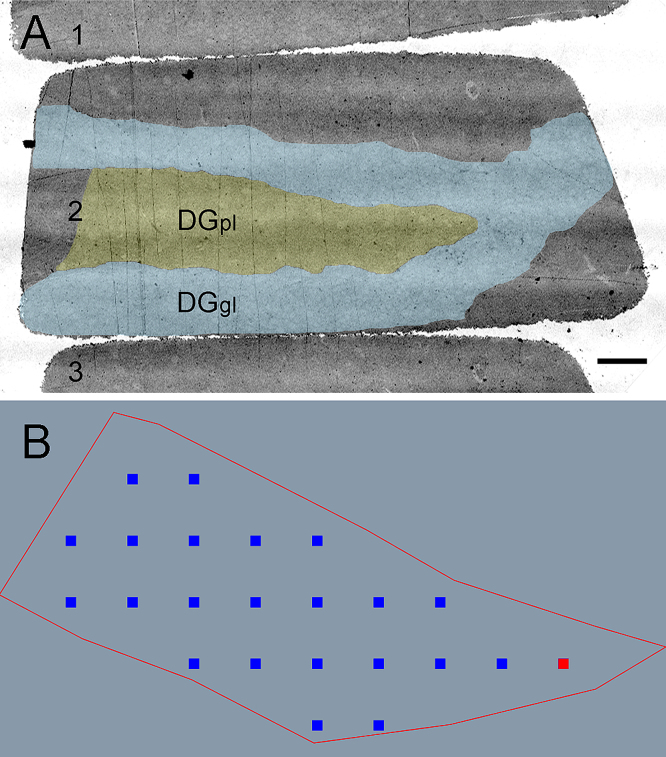
Defining the region of interest. (A) Electron micrograph of serial thin sections numbered 1–3 showing the polymorph cell layer of the dentate gyrus (DGpl) and the surrounding granular cell layer (DGgl). (B) A grid of seeds for images (blue squares, one seed is highlighted as a red square) is displayed within the border of the dentate gyrus (red lines) by our custom-made software. Scale bar 30 μm in A. (For interpretation of the references to color in this figure legend, the reader is referred to the web version of this article.)

Thereafter, the coordinates of edge points forming a polygon that approximates the border of the DGpl were fed into the custom-made software. The border was visible using low magnification EM (170×, Fig. 2A) and confirmed by comparing the electron microscopic image with a toluidine blue-stained semi-thin parallel section taken from the same block (see Fig. 1C). The microscope stage was manually moved to outline the DGpl as a polygon, which was then recorded by the software.

Next, the software calculated at least 20 seeds for the positions of the micrographs within the boundaries of the outlined area. For this, a regular grid of seeds was produced (Fig. 2B) with an adjustable distance between the seeds. The x and y shift of the grid were randomly determined using the pseudo-random number generator Math.Random provided by Java Script. The Points-In-Polygon algorithm was used to select only those seeds which were within the polymorph layer boundaries, using a ray crossing algorithm that refers to the Jordan Curve Theorem (Shimrat, 1962; Haines, 1994). Each of the 20 seeds was used to control the microscope's *x* and *y* stage shifts, and an image was taken at each position using the FEI Montage software to merge four adjacent fields of view taken at a magnification of 5000× each. The FEI Serial Section software permitted to go from the position on the first section, the reference section, to a corresponding position on the second section, the lookup section. The shift was manually corrected and the second image of the pair was made in the lookup section using Montage software.

**Fig. 3:**
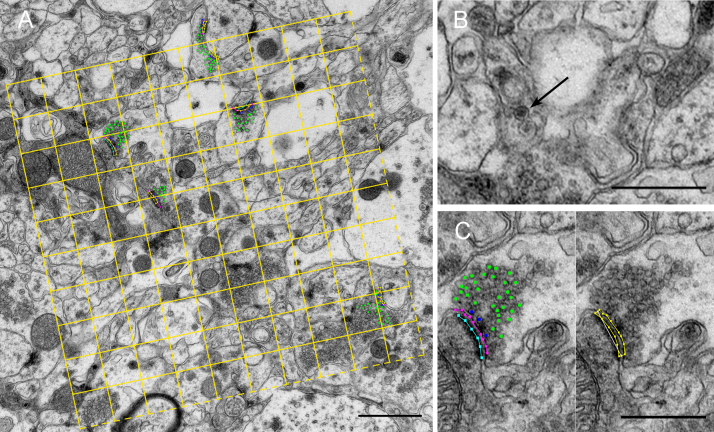
Measuring ultrastructural parameters with an unbiased counting frame. (A) An unbiased counting frame subdivided by auxiliary lines was used to determine synapse numbers and the numbers of DCVs. (B) The arrow depicts a DCV. (C) Left panel: the length of the presynaptic membrane cross section (pink line), the postsynaptic density cross section (turquoise line), and the numbers of docked (blue dots) and undocked (green dots) synaptic vesicles were measured on single sections. (C) Right panel: the mean width of the synaptic cleft was determined by dividing the area of the polygon (yellow line) by the length of the central line (white). Scale bars 1 μm in A, 500 nm in B and C.

A disector was used on the image pairs for assessing the numbers of synapses and DCV. For this, the section pairs were aligned using ImageJ software. A 5.5 μm × 5.5 μm counting frame was placed at a random position and random angle within the image's boundaries (Fig. 3A), onto each reference section and at the corresponding position of each lookup section using custom-made software. The counting frame was subdivided with auxiliary lines to facilitate counting of synapses and DCV as well as the evaluation of ultrastructural parameters. The pseudo random number generator provided by Java.util was used for determining random positions and angles. Synapses and DCV (Fig. 3B) were only counted if they were present within the outer borders of the counting frame on the reference sections, but not on the lookup sections. They were also neglected if they went across the top or right border of the counting frame. Synapse counts and counts of DCV were derived from all 20 image pairs and the mean synapse count/counting frame of each animal was used for statistical analysis.

Furthermore, several ultrastructural parameters were determined for each animal: the average length of the presynaptic membrane cross section and of the postsynaptic density in the cross section, the average width of the synaptic cleft as well as the average number of docked vesicles (vesicles with a maximum distance from the presynaptic membrane of one vesicle diameter) and undocked vesicles (those with a maximum distance of one vesicle diameter from docked or other undocked vesicles at the same synapse) within the thin section (Fig. 3C, left panel). The mean width of the synaptic cleft was approximated from the area of the polygon shown in Fig. 3C (right panel) divided by the length of the central line (Fig. 3C, right panel). For this, all synapses present on the reference section with a synaptic cleft visible in the cross section were selected. Between 19 and 29 synapses were found in each animal. The ObjectJ platform of ImageJ was used to measure these parameters. The mean values of the measurements on these synapses were used for statistics. All measurements and counts were made on coded samples by an experimenter blind to the treatment groups.

### 2.5 Statistics

Statistical evaluation of the results was performed using SPSS 20.0 (SPSS Inc., Chicago, IL, USA). Ultrastructural data were analyzed by Student's *t*-test. For these data, extreme values more than 3 interquartile ranges above the 75% percentile or below the 25% percentile were excluded from the analysis. Behavioral data were analyzed with two-way repeated-measures ANOVA (within-subject factor: test day, between-subject factor: housing condition). The homogeneity of variances was assessed with the Levene test. Post hoc ANOVA analysis of test day differences was performed with the Games-Howell test, as the variances were unequal. Housing condition differences at each test day were analyzed with the Mann–Whitney-*U* test. The Bonferroni correction was used to adjust for multiple testing. Probability values ≤0.05 were regarded as statistically significant. All data are presented as means ± SEM, *n* referring to the number of mice in each group.

## 3 Results

### 3.1 Environmental enrichment improves spatial learning and memory in the Morris water maze

**Fig. 4:**
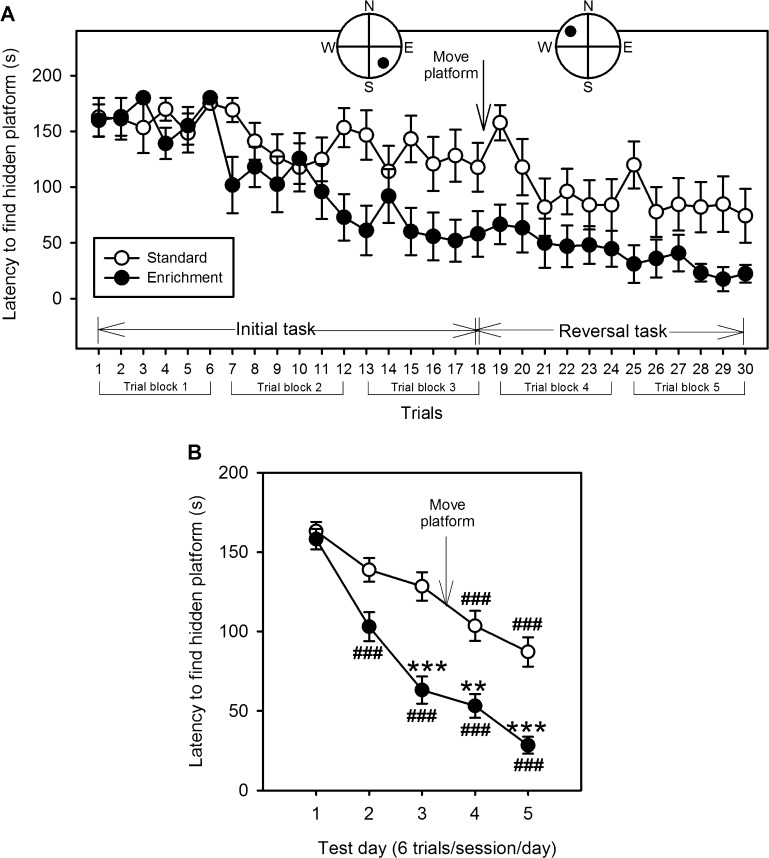
Environmental enrichment (EE) improves spatial learning and memory. Panel A describes the average latency of standard and EE-housed mice to find the hidden platform in the Morris water maze. Animals were subjected to 6 trials/day on 5 consecutive days (trial blocks 1–5). The insert describes the location of the platform within the tank. For the first 3 days the platform was located in the southeast quadrant of the tank (initial task), after which it was moved to the northwest quadrant (reversal task). As indicated in panel B, EE-housed animals were faster in locating the hidden platform than standard-housed mice from test day 3 onwards. Both groups performed better after multiple trials. **P < 0.01, ***P < 0.001 vs. standard-housed animals; ^###^*P* < 0.001 vs. treatment day 1 of the respective housing condition. The values represent means ± SEM, *n* = 10/group.

In the MWM we assessed the ability of mice to locate a hidden platform in a water-filled tank and especially their ability to remember the location of the platform upon repeated trials, which is reflected by a shorter latency to reach the platform. Two-way repeated-measures ANOVA revealed that the latency to find the platform in EE-housed mice was significantly shorter than in standard-housed mice (*F*_(1, 116)_ = 56.06, *P* < 0.001), indicating that spatial learning/memory was improved by EE. In addition to housing condition, the latency also differed with trial day (*F*_(3.54, 410.54)_ = 56.54, *P* < 0.001), with a significant interaction between these factors (*F*_(3.54, 410.54)_ = 5.40, *P* = 0.001). As depicted in Fig. 4A, repeated trials shortened the latency to find the hidden platform in both groups, and repositioning of the platform (reversal task) did not increase the latency for either group. As shown in Fig. 4B on test day 3 (*P* < 0.001), 4 (*P* < 0.01) and 5 (*P* < 0.001), EE-housed mice were significantly faster in finding the hidden platform in the tank. Furthermore both groups improved in their ability to locate the hidden platform after multiple trials. However, compared to test day 1 EE-housed animals performed significantly better from test day 2 onwards (*P* < 0.001), while standard-housed mice performed better only from test day 4 onwards (*P* < 0.001).

### 3.2 Environmental enrichment has distinct effects on the ultrastructure of the dentate gyrus

**Fig. 5:**
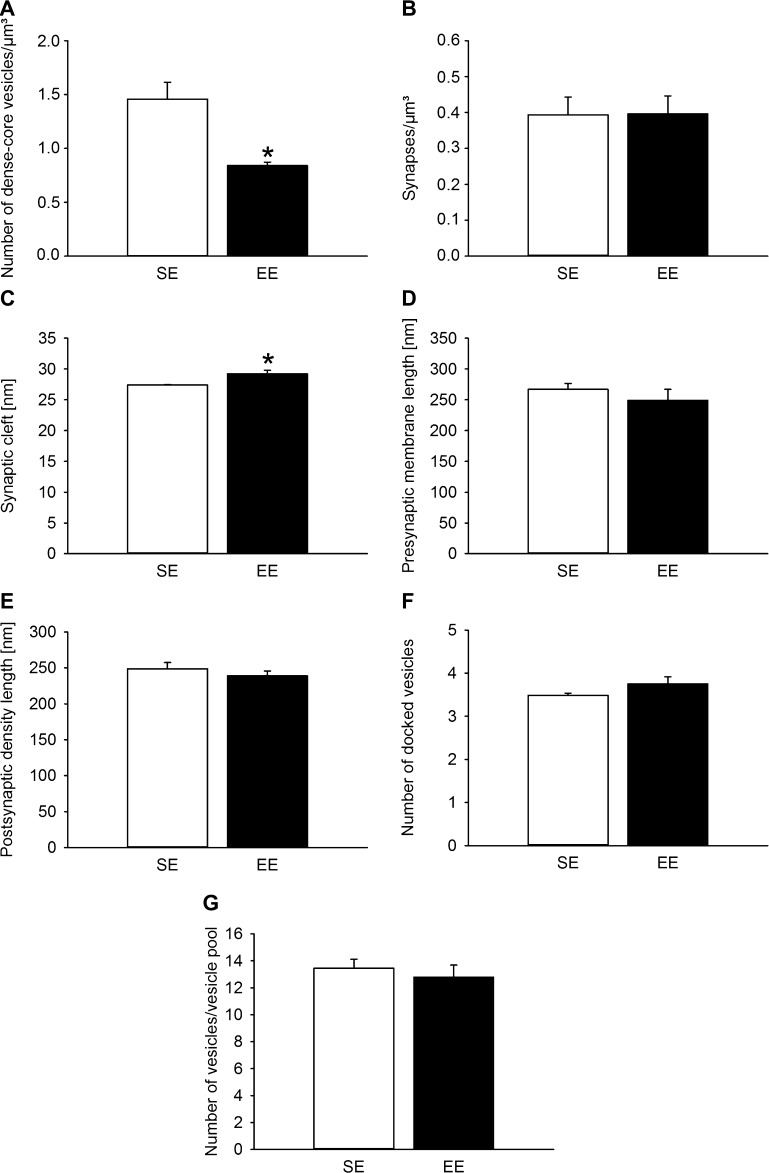
Environmental enrichment decreases the number of dense-core vesicles (A) and increases the width of the synaptic cleft (C) in the polymorph cell layer of the dentate gyrus. The number of synapses/μm^3^ (B), presynaptic membrane length, postsynaptic density length, number of docked vesicles and number of vesicles in the vesicle pool were not affected by the differential housing conditions (D–G). The ultrastructure of the polymorph cell layer of the dentate gyrus was assessed in mice kept in standard environment (SE) or environmental enrichment (EE) for 5 weeks. *P < 0.05 vs. SE. The values represent means + SEM, A, E, and F: *n* = 4–5/group; B, D, and G: *n* = 5/group; C: *n* = 3–5/group.

Statistical analysis of parameters determined using a disector revealed that the number of DCV per μm^3^ within the DGpl was significantly lower (*P* < 0.05) in EE-housed animals (Fig. 5A) than in standard-housed animals, but the number of synapses per μm^3^ did not differ (Fig. 5B).

Of those parameters determined on single sections, environmental enrichment significantly increased the average width of the synaptic cleft (*P* < 0.05; Fig. 5C). In contrast, other measures of the synaptic ultrastructure including presynaptic membrane length, postsynaptic density length, number of docked clear vesicles and number of clear vesicles in the vesicle pool did not change after the differential housing period (Fig. 5D–G).

## 4 Discussion

Here we tested a novel unbiased sampling protocol to assess the numerical density of synapses and DCV in the brain with EM. Our procedure allows the examination of any subregion (nuclei, defined cell layers) of the brain by EM with high accuracy. Furthermore, the micrographs were taken at predetermined positions within the target structure rather than arbitrarily distributing them through the area of interest. A random number generator was used to determine these positions, ensuring systematic and random sampling. This has enabled us to detect significant changes in the numerical densities of DCV and synaptic cleft diameters in the DGpl of mice that had been kept under EE.

We confirm previous results that the numerical density and ultrastructural features of synapses can change during EE, indicating changes in neuronal activity due to this enriched housing condition (Nakamura et al., 1999; Rampon et al., 2000; Xu et al., 2009; Landers et al., 2011; Lonetti et al., 2010). Depending on the brain area examined and the enrichment protocol used, previous studies described manifold effects of EE on neuropil ultrastructure: when juvenile rats were maintained under EE conditions, an increased synaptic vesicle content was reported in the parietal cortex (Nakamura et al., 1999). Juvenile EE was also reported to modify numerical synapse densities in the cerebellum and the primary somatosensory cortex of mice (Lonetti et al., 2010). When EE was provided during adulthood, synapse numbers increased in the barrel cortex (Landers et al., 2011) and in the CA1 region of the hippocampus (Rampon et al., 2000). Furthermore, ultrastructural modifications of synaptic junctions were found in CA1 and the parietal cortex (Xu et al., 2009).

Care must be taken when comparing these previously published results with each other and with our findings: not only were different EE protocols used and the investigated brain regions differed from study to study, the results were also obtained with different methods. For instance, serial section reconstructions were used to quantify the synapses in the mouse barrel cortex by Landers et al. (2011), a very accurate but time consuming procedure. Nakamura et al. (1999) estimated synapse numbers from single sections, which is prone to overestimation bias when the aim is to determine the numerical density of synapses within a defined volume (Howard and Reed, 2004). In contrast, unbiased quantification using disectors was applied in several other EE studies to assess the numerical synapse densities (Rampon et al., 2000; Xu et al., 2009; Lonetti et al., 2010). We adapted previous techniques so that numerical synapse density, numerical DCV density, and other ultrastructural parameters could be determined using the disector technique in the DGpl of the mouse. One challenge of EM experiments using stereology within small brain areas like the DGpl is ensuring comparability of selected brain coordinates between each experimental animal. We overcame this challenge by approaching the dentate gyrus on only 20 μm thin vibratome sections, significantly thinner than in previously published reports (Morshedi et al., 2009). Novel to our procedure was that the vibratome sections were Nissl-stained so that they were easy to compare with the Nissl-stained micrographs depicted in the mouse brain atlas of Franklin and Paxinos (2008). Once the desired level of the dentate gyrus was identified, an adjacent section was taken, microdissected and processed for EM. After trimming the blocks, the areas of interest were immediately present on the block surface. This presented an appreciable advantage in our hands when compared with the identification of the respective brain areas on samples that had been microdissected and embedded without prior production of vibratome sections (data not shown). In samples that had been embedded without prior production of vibratome sections, it took several hours to find the areas of interest.

Another, general challenge in stereology is that an unbiased sampling protocol needs to be implemented (Howard and Reed, 2004). This seems difficult to adhere to in classical electron microscopic studies where the EM operator had to move the stage by hand with the risk of introducing bias. In previous studies, the samples were either spread over the area of interest (Rampon et al., 2000), or every image pair was taken at regular intervals of two widths of the EM screen from previous pairs (Morshedi et al., 2009; Hunter and Stewart, 1993; Ingham et al., 1998; Day et al., 2006). We overcame this challenge by automatically controlling the stage shifts with custom-made software, calculating the initial shifts with a random number generator, thus avoiding human bias when controlling the stage. Random numbers were used before to obtain EM samples in a stereological analysis (Morshedi et al., 2009), but novel to our study is that random numbers were used to control microscope stage shifts.

Our automatic stage shift procedure did not save time as compared to moving the stage by hand (data not shown). In both cases the outlines of the neuropil under study had to be defined before the micrographs were made – before automatically moving the stage, the area of interest had to be defined using custom-made software, whereas in control experiments, the FEI user interface was used to define this area, which took approximately the same time. However, automatically moving the stage to shifts determined with a random number had the advantage that no operator bias was introduced.

Whereas our protocol excluded observer bias from the sampling procedure, our custom-made software did not replace the software made by the electron microscope's supplier. TEM Image Analysis, Serial Section, and Montage software provided by FEI were all used in addition to custom-made software, which may have been a disadvantage as one operator had to use several different software systems sequentially.

An important aspect of this study was to confirm that the enrichment protocol used can influence behavior and thus brain function of mice increasing the likelihood to detect morphological changes. This was done by the use of the MWM, a hippocampus-dependent behavioral test for spatial learning and memory (Sharma et al., 2010). The enhanced performance of EE-housed mice in this test observed here is in line with previous data from our group and from other studies (Reichmann et al., 2013; Williams et al., 2001; Harburger et al., 2007).

For the first time we found an influence of EE on the DCV number within the brain. Specifically, EE-housed animals had a significantly lower numerical density of DCV in the DGpl than control animals. DCV are storage sites for neuropeptides. One major component of DCV in the DGpl is likely to be NPY. NPY is one of the most abundant neuropeptides in the brain (Tatemoto et al., 1982), and it is known to be synthesized in the DGpl (Kask et al., 2002). The biological actions of NPY include effects on emotional-affective behavior, stress resilience and cognition (Heilig, 2004; Cohen et al., 2012). EE has been shown to increase NPY mRNA expression in the DGpl (Reichmann et al., 2012), and we show here that EE has a significant effect on spatial learning and memory. We thus speculate that NPY may be one mediator of the effects of EE in the DGpl. The decrease in the numerical density of DCV after EE signifies that EE reduces the amount of neuropeptides stored at the time of fixation. The increase in NPY mRNA expression due to EE (Reichmann et al., 2012) and the behavioral effects seen in this study may reflect an increased release of NPY caused by EE. NPY expression may be upregulated to keep up with demand and at the same time the number of storage vesicles may be reduced. Further studies will be necessary to confirm this hypothesis.

We also show that EE significantly increased the width of the synaptic clefts within the DGpl. This finding is difficult to interpret as previous toxicological studies suggested widened synaptic clefts as a morphological correlate of impaired brain function (Jing et al., 2004; Han et al., 2014). However, our current results show that enhanced cleft width of EE-housed mice within the DGpl is associated with a beneficial effect of EE in the MWM, a hippocampus-dependent memory task. Thus, an alternative explanation may consider the neuronal plasticity of the brain in response to different stimuli. Remodeling of synapses is a process taking place throughout life involving continuous strengthening and weakening of synaptic contacts depending on the environmental needs. Thus a widened synaptic cleft may also be regarded as a physiological adaptation process following enhanced stimulation. Further studies, if feasible with high pressure freezing rather than chemical fixation, would be necessary to confirm this hypothesis.

While we found significant changes in the number of DCV and in the width of the synaptic clefts, we did not detect changes in the numerical density of synapses or in several other synaptic parameters between mice that had been housed under EE or standard conditions. This is the first report of stereological synapse evaluation within the DGpl in an enrichment setting, so it cannot be directly compared to other studies of other brain regions with similar settings. As mentioned above, EE effects on the synaptic ultrastructure depend heavily on brain region examined and EE paradigm used (Nakamura et al., 1999; Rampon et al., 2000; Xu et al., 2009; Landers et al., 2011; Lonetti et al., 2010).

In summary, we devised an unbiased sampling protocol suitable for assessing ultrastructural parameters of synapses in very small brain areas. Our approach will be especially useful to investigate small brain regions like the DGpl with a precision of a few micrometers. Human bias was excluded from sample analysis because the stage shifts were automatically controlled.
